# Prognostic Value of the Pretreatment Lung Immune Prognostic Index in Advanced Small Cell Lung Cancer Patients Treated With First-Line PD-1/PD-L1 Inhibitors Plus Chemotherapy

**DOI:** 10.3389/fonc.2021.697865

**Published:** 2021-10-08

**Authors:** Lingling Li, Chenghui Pi, Xin Yan, Jiangyue Lu, Xuhui Yang, Chunyu Wang, Xiaoyan Li, Sujie Zhang, Zhibo Zhang, Yi Sun, Yi Hu

**Affiliations:** ^1^ School of Medicine, Nankai University, Tianjin, China; ^2^ Department of Oncology, The First Medical Center of Chinese People’s Liberation Army (PLA) General Hospital, Beijing, China; ^3^ Department of Further Education, Harbin Medical University Cancer Hospital, Harbin, China; ^4^ Department of Cardiothoracic Surgery, The 78th Group Army Hospital of Chinese PLA, Mudanjiang, China; ^5^ State Key Laboratory of Transducer Technology, Shanghai Institute of Microsystem and Information Technology, Chinese Academy of Sciences, Shanghai, China

**Keywords:** small cell lung cancer, immune checkpoint inhibitor, first-line, lung immune prognostic index, prognosis

## Abstract

**Background:**

Lung immune prognostic index (LIPI) refers to a biomarker combining derived neutrophil-to-lymphocyte ratio (dNLR) and lactate dehydrogenase (LDH). Its prognostic effect on advanced small cell lung cancer (SCLC) patients receiving programmed cell death 1/programmed cell death ligand-1 (PD-1/PD-L1) inhibitors plus chemotherapy as first-line treatment remains unclear. Our research investigated the relationship between pretreatment LIPI and the prognosis of patients receiving first-line PD-1/PD-L1 inhibitors plus chemotherapy.

**Methods:**

Advanced SCLC patients receiving PD-1/PD-L1 inhibitors plus chemotherapy as first-line treatment from Jan 2015 to Oct 2020 were included. Based on the values of dNLR and LDH, the study population was divided into two groups: LIPI good and LIPI intermediate/poor. The Kaplan-Meier method was used to compute the median survival time and the log-rank test was used to compare the two groups. Univariate and multivariate analyses were used to examine the correlation between the pretreatment LIPI and clinical outcomes.

**Results:**

One hundred patients were included in this study, of which, 64% were LIPI good (dNLR < 4.0 and LDH < 283 U/L), 11% were LIPI poor (dNLR ≥ 4.0 and LDH ≥ 283 U/L), and the remaining 25% were LIPI intermediate. The LIPI good group had better progression-free survival (PFS) (median: 8.4 *vs* 4.7 months, *p* = 0.02) and overall survival (OS) (median: 23.8 *vs* 13.3 months, *p* = 0.0006) than the LIPI intermediate/poor group. Multivariate analysis showed that pretreatment LIPI intermediate/poor was an independent risk factor for OS (HR: 2.34; 95%CI, 1.13, 4.86; *p* = 0.02). Subgroup analysis showed that pretreatment LIPI good was associated with better PFS and OS in males, extensive disease (ED), PD-1 inhibitor treatment, smokers, and liver metastasis (*p* < 0.05).

**Conclusions:**

Pretreatment LIPI could serve as a prognostic biomarker for advanced SCLC patients receiving first-line PD-1/PD-L1 inhibitors plus chemotherapy.

## Introduction

Small-cell lung cancer (SCLC) constitutes 13 - 15% of total lung cancer cases, and is characterized by rapid progression and early distant metastasis (1, 2). Over 90% of SCLC patients are elders or past heavy smokers (3). One-third of SCLC patients are classified as having limited disease (LD), and the others as having extensive disease (ED) according to the Veteran’s Administration Lung Cancer Study Group Staging System (4, 5). Despite sensitivity to first-line chemotherapy, most SCLC cases recur in one year and are insensitive to second-line treatment (6). The median overall survival (OS) is 15−20 months for patients with LD, and 8−13 months for those with ED (7).

Immune checkpoint inhibitors (ICIs), especially programmed cell death 1/programmed cell death ligand-1 (PD-1/PD-L1) inhibitors, have revolutionized the treatment landscape of various cancers. Recently, the IMpower 133 and CASPIAN studies have demonstrated that a combination of atezolizumab or durvalumab and chemotherapy could improve clinical outcomes of SCLC patients as compared to those using chemotherapy alone (8, 9). The phase II EA5161 study has demonstrated the addition of nivolumab at first-line treatment significantly improved the progression-free survival (PFS) and OS of ES-SCLC patients (median PFS: 5.5 *vs* 4.6 months, *p* = 0.012; median OS: 11.3 *vs* 8.5 months, *p* = 0.038) (10). The phase III KEYNOTE-604 study showed that advanced SCLC patients receiving first-line pembrolizumab plus chemotherapy had better OS compared with those receiving chemotherapy alone, but the difference did not meet the predefined statistical threshold (11). A meta analysis study found that both PD-L1 inhibitors and PD-1 inhibitors plus chemotherapy as first-line treatment could provide a significant improvement of survival time compared with chemotherapy alone for advanced SCLC patients (12). FDA has approved PD-1 inhibitors as third-line treatment in 2018 and PD-L1 inhibitors as first-line treatment in 2020 for patients with ED or relapsed SCLC, which is an important advancement for SCLC patients.

SCLC patients have a relatively high tumor mutation burden (13), but it has not been proven to serve as a clear predictor in patients receiving ICI treatment (8, 14). PD-L1 expression is low or absent in SCLC patients, but it is still not used as a predictive biomarker in SCLC patients receiving ICI treatment (15). Currently, no prognostic biomarkers can definitely guide the application of ICIs in patients with SCLC. Therefore, identifying biomarkers to select patients who are likely to respond to immunotherapy is crucial. Systemic inflammation plays a critical role in the occurrence and development of cancer (16). Previous studies have reported the prognostic role of systemic inflammation indicators in non-small cell lung cancer (NSCLC) patients receiving immunotherapy, including neutrophil-to-lymphocyte ratio (NLR) and lactate dehydrogenase (LDH) (17–22). Several studies showed that the lung immune prognostic index (LIPI), combining derived NLR (dNLR, absolute neutrophil count/[white blood cell concentration−absolute neutrophil count]) and LDH, could predict survival in advanced NSCLC patients receiving immunotherapy (23, 24). However, there is a lack of studies describing the prognostic value of pretreatment LIPI in advanced SCLC patients receiving PD-1/PD-L1 inhibitor treatment. Therefore, we aim to investigate whether pretreatment LIPI was related to the prognosis of advanced SCLC patients treated with first-line PD-1/PD-L1 inhibitors plus chemotherapy.

## Methods

### Study Design and Patients

The study was carried out at the Chinese PLA general hospital (Beijing, China). Advanced SCLC patients receiving PD-1/PD-L1 inhibitors plus chemotherapy as first-line treatment from Jan 2015 to Oct 2020 were included. The inclusion criteria were as follows (1): patients who were diagnosed with SCLC (2); patients receiving first-line PD-1/PD-L1 inhibitors plus chemotherapy; and (3) patients who were treated with at least two cycles of PD-1/PD-L1 inhibitors. The exclusion criteria were (1): absence of efficacy assessment; and (2) absence of pretreatment blood test results. Clinical characteristics as well as pretreatment blood laboratory test results were recorded. Clinical characteristics included age, sex, stage, smoking history, ICI drugs, Eastern Cooperative Oncology Group Performance Status (ECOG PS), sites of metastasis and efficacy, and pretreatment blood test results included total white blood cell count, absolute neutrophil count, absolute lymphocyte count, and LDH levels. This research was authorized by the Ethics Committee of Chinese PLA General Hospital and performed according to the principles of the Declaration of Helsinki.

LD is defined as a disease limited to one hemithorax, local mediastinal lymph nodes, and ipsilateral supraclavicular lymph nodes, which can be included in a tolerable radiation field; ED includes the cases not classified as LD (25). Blood tests were conducted within 5 days before the first cycle of immunotherapy. LIPI was calculated by dNLR (absolute neutrophil count/[white blood cell concentration−absolute neutrophil count]) and LDH, and cutoff values of dNLR and LDH were calculated using X-tile software based on data (26), which were 4.0 U/L and 283 U/L, respectively. Patients were stratified into LIPI good (dNLR < 4.0 and LDH < 283 U/L) and LIPI intermediate/poor groups (intermediate: dNLR < 4.0 and LDH ≥ 283 U/L, or dNLR ≥ 4.0 and LDH < 283 U/L; poor: dNLR ≥ 4.0 and LDH ≥ 283 U/L) groups.

Treatment responses were assessed every two cycles of ICI treatment by two independent investigators (ZZ and LL) according to the Response Evaluation Criteria in Solid Tumors (RECIST) version 1.1, including complete response (CR), partial response (PR), stable disease (SD), and progressive disease (PD). PFS was defined as the period from the first ICI treatment to disease progression or death (whichever occurred first). OS was defined as the period from the first ICI treatment to death. All patients were followed up through telephone counseling and searching electronic medical records with a cutoff date of March 16, 2021.

### Statistical Analysis

Statistical analyses were conducted using IBM SPSS 19.0 (SPSS Inc., Chicago, IL, USA) and GraphPad Prism 8 (La Jolla, CA, USA). X-tile 3.6.1 software (Yale University, New Haven, CT, USA) was used to identify the optimal cut-off values for dNLR and LDH. Kaplan-Meier curves were used to analyze OS and PFS, and the differences were evaluated by log-rank test. Chi-square or Fisher’s exact test was used to compare categorical variables. Hazard ratio (HR) with its 95% confidence interval (CI) was estimated by Cox proportional hazards models. Univariate and multivariate analyses were conducted to determine the independent prognostic value of pretreatment LIPI. The variables with *p* < 0.05 in the univariate analysis were eligible to be included in the multivariate analysis. Phi correlation coefficients were calculated to determine the association between each pair of the dichotomous variables. All statistical tests were two-sided with a statistical significance of *p* < 0.05.

## Results

### Patient Clinical Characteristics

**Figure 1:**
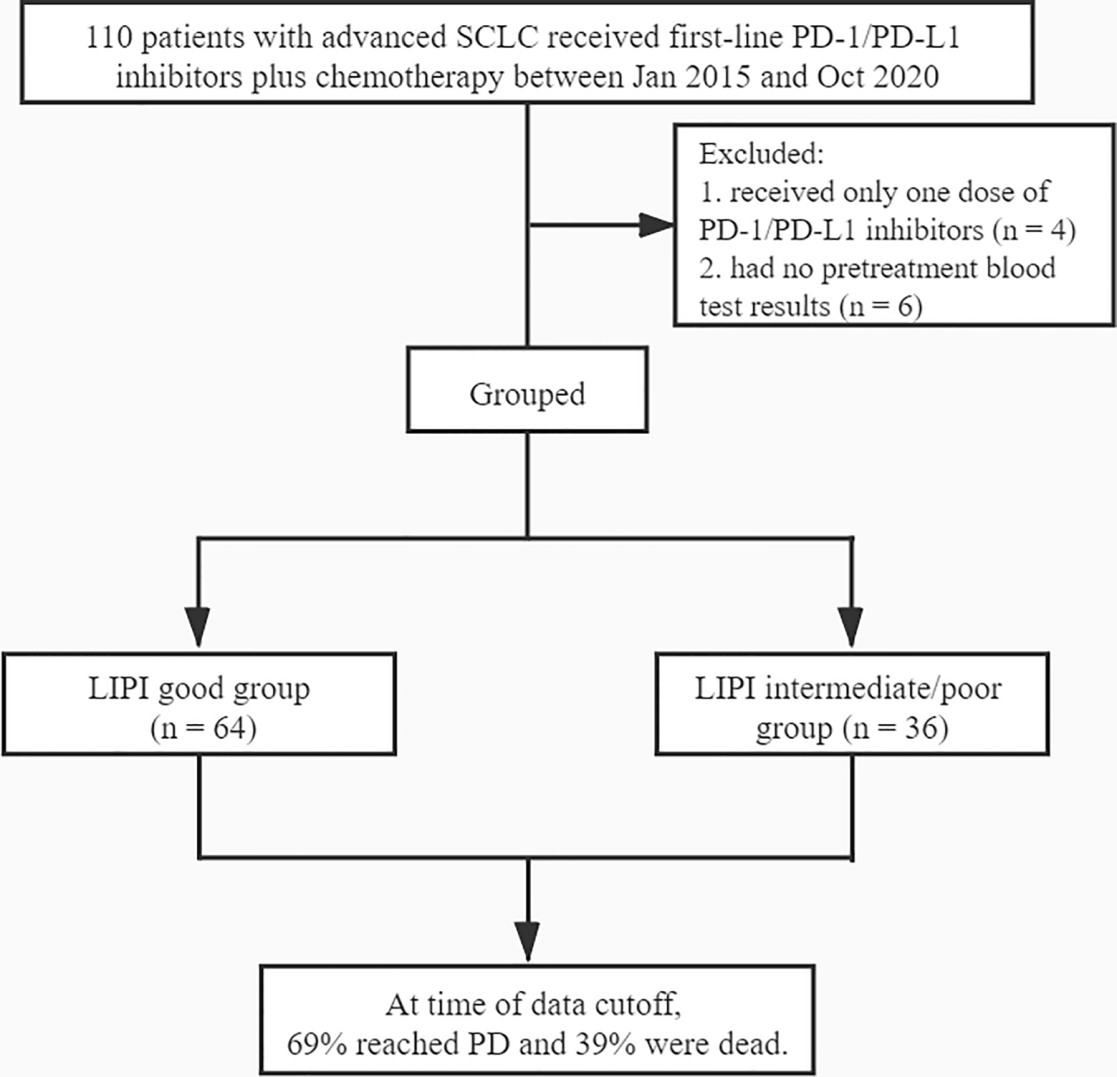
Diagram of the study.

A total of 110 SCLC patients receiving first-line PD-1/PD-L1 inhibitors were identified, of which, four patients received only one dose of PD-1/PD-L1 inhibitors, and six patients had no pretreatment blood test results (**Figure 1**). Finally, 100 SCLC patients were included for data analysis. Most of those patients (87%) received platinum-etoposide chemotherapy (45% carboplatin and 42% cis-platinum), and the other patients (13%) received nab-paclitaxel and etoposide. Moreover, 65% of the patients received PD-1 inhibitors (nivolumab, pembrolizumab or sintilimab), and 35% received PD-L1 inhibitors (atezolizumab or durvalumab). Patients had a maximum of 4-6 cycles of chemotherapy as first-line treatment. The median follow-up time was 19.2 months. Detailed clinical data of the patients are summarized in **Table 1**. The median age was 60 years (range: 32−82). Among the 100 patients, 88% were males, 74% had an ED, 94% had an ECOG PS of 0−1, 79% had a smoking history, 22% had brain metastasis, 24% had liver metastasis, and 29% had bone metastasis. Of the patients, 60%, 31%, and 9% had PR, SD, and PD, respectively; 78% had dNLR < 4.0, and 75% had LDH < 283 U/L. Patients in the LIPI good, LIPI intermediate, and LIPI poor groups were 64%, 25%, and 11%, respectively.

**Table 1:** Characteristics of patients with advanced SCLC.

Characteristics	No. of patients (n = 100)	Percentage (%)
Age (year), median (range)	60 (32−82)	
<60	48	48
≥60	52	52
Sex		
Male	88	88
Female	12	12
Stage		
LD	26	26
ED	74	74
Smoking history		
Never smoke	21	21
Smoke	79	79
ICI Drugs		
PD-1 inhibitor	65	65
PD-L1 inhibitor	35	35
Chemotherapy		
Platinum plus etoposide	87	87
Nab-paclitaxel plus etoposide	13	13
ECOG PS		
0−1	94	94
≥2	6	6
Brain metastasis		
Yes	22	22
No	78	78
Liver metastasis		
Yes	24	24
No	76	76
Bone metastasis		
Yes	29	29
No	71	71
Treatment efficacy		
PR	60	60
SD	31	31
PD	9	9
dNLR		
<4.0	78	78
≥4.0	22	22
LDH (U/L)		
<283	75	75
≥283	25	25
Pretreatment LIPI		
Good	64	64
Intermediate	25	25
Poor	11	11

LD, limited disease; ED, extensive disease; ECOG PS, Eastern Cooperative Oncology Group Performance Status; ICI, immune checkpoint inhibitor; PD-1, programmed cell death-1; PD-L1, programmed cell death-ligand 1; dNLR, derived neutrophil-to-lymphocyte ratio; LDH, lactate dehydrogenase; LIPI, Lung immune prognostic index; PR, partial response; SD, steady disease; PD, progressive disease.

### Univariate and Multivariate Analysis for PFS and OS

**Figure 2:**
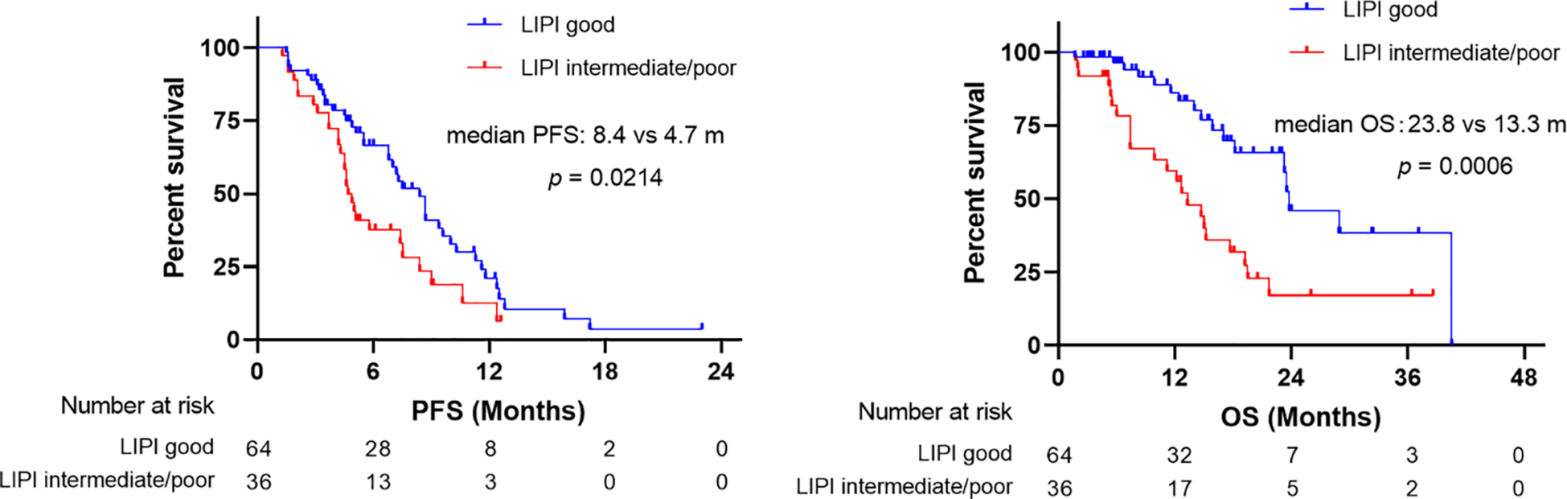
Association between pretreatment LIPI with PFS and OS.

At time of data cutoff, 69% of the patients reached PD and 39% died. LIPI good was associated with better PFS than LIPI intermediate/poor (median: 8.4 *vs* 4.7 months, *p* = 0.02) (**Figure 2**). Univariate analysis demonstrated that ECOG PS 0−1, no bone metastasis, and pretreatment LIPI good were related to better PFS in SCLC patients receiving first-line ICI treatment (*p* < 0.05). Before multivariate analysis, the pairwise correlation coefficients of ECOG PS, bone metastasis, and pretreatment LIPI were calculated to determine the potential correlation between each pair of these variables. All the correlation coefficients were below 0.5, indicating that there was a low correlation between each pair of these variables (**Table 2**). After multivariate analysis, the results indicated that ECOG PS ≥ 2 (HR: 2.58; 95%CI, 1.10, 6.04; *p* = 0.03) and bone metastasis (HR: 2.53; 95%CI, 1.47, 4.37; *p* = 0.001) were independent risk factors for PFS. In contrast, pretreatment LIPI intermediate/poor (HR: 1.42; 95%CI, 0.84, 2.39; *p* = 0.19) was not an independent risk factor for PFS in multivariate analysis (**Table 3**).

**Table 2:** Correlation coefficient between each pair of the variables selected by univariate analysis.

Correlation coefficients	ECOG PS	Bone metastasis	Pretreatment LIPI	ICI drugs	Stage	Liver metastasis
ECOG PS	–	0.117	0.161	-0.185	0.150	0.351
Bone metastasis	0.117	–	0.347	0.085	0.379	0.415
Pretreatment LIPI	0.161	0.347	–	-0.026	0.160	0.310
ICI drugs	-0.185	0.085	-0.026	–	-0.043	0.128
Stage	0.150	0.379	0.160	-0.043	–	0.333
Liver metastasis	0.351	0.415	0.310	0.128	0.333	–

ECOG PS, Eastern Cooperative Oncology Group Performance Status; LIPI, lung immune prognostic index; ICI, immune checkpoint inhibitor.

**Table 3:** Univariate and multivariate analysis for PFS in SCLC patients treated with ICIs.

Variable	Category	Univariate analysis		Multivariate analysis	
		HR (95% CI)	*p-value*	HR (95%CI)	*p-value*
Age (year)	≥60 *vs* <60	1.14 (0.71, 1.84)	0.59	—	—
Sex	Female *vs* Male	1.28 (0.63, 2.58)	0.50	—	—
Smoking history	Yes *vs* No	0.58 (0.33, 1.02)	0.06	—	—
ICI drugs	PD-L1 inhibitors *vs* PD-1 inhibitors	1.35 (0.82, 2.22)	0.24	—	—
Stage	ED *vs* LD	0.99 (0.57, 1.72)	0.98	—	—
ECOG PS	≥2 *vs* 0−1	2.71 (1.16, 6.35)	0.02	2.58 (1.10, 6.04)	0.03
Brain metastasis	Yes *vs* No	1.13 (0.64, 1.98)	0.68	—	—
Liver metastasis	Yes *vs* No	1.57 (0.91, 2.69)	0.10	—	—
Bone metastasis	Yes *vs* No	2.81 (1.67, 4.73)	<0.001	2.53 (1.47, 4.37)	0.001
Pretreatment LIPI	Intermediate/Poor *vs* Good	1.76 (1.08, 2.89)	0.03	1.42 (0.84, 2.39)	0.19

ICI, immune checkpoint inhibitor; PD-1, programmed cell death-1; PD-L1, programmed cell death-ligand 1; LD, limited disease; ED, extensive disease; ECOG PS, Eastern Cooperative Oncology Group Performance Status; LIPI, lung immune prognostic index; HR, hazard ratio; CI, confidence interval.

As shown in **Figure 2**, patients with LIPI good had better OS than those with LIPI intermediate/poor (median: 23.8 *vs* 13.3 months, *p* = 0.0006). Univariate analysis showed that PD-1 inhibitor treatment, LD, ECOG PS 0−1, no liver metastasis, no bone metastasis, and pretreatment LIPI good were related to better OS (*p* < 0.05). All the pairwise correlation coefficients of ICIs drugs, stage, ECOG PS, liver metastasis, bone metastasis, and pretreatment LIPI were below 0.5 (**Table 2**). After multivariate analysis, the results showed that PD-L1 inhibitors (HR: 2.37; 95%CI, 1.10, 5.11; *p* = 0.03), ECOG PS ≥ 2 (HR: 6.96; 95%CI, 2.25, 21.55; *p* = 0.001), liver metastasis (HR: 2.66; 95%CI, 1.19, 5.93; *p* = 0.02), bone metastasis (HR: 4.61; 95%CI, 2.01, 10.59; *p* < 0.001), and LIPI intermediate/poor (HR: 2.34; 95%CI, 1.13, 4.86; *p* = 0.02) were independent risk factors for OS (**Table 4**).

**Table 4:** Univariate and multivariate analysis for OS in SCLC patients treated with ICIs.

Variable	Category	Univariate analysis		Multivariate analysis	
		HR (95% CI)	*p-value*	HR (95%CI)	*p-value*
Age (year)	≥60 *vs* <60	1.22 (0.65, 2.32)	0.54	—	—
Sex	Female *vs* Male	0.34 (0.08, 1.40)	0.13	—	—
Smoking history	Yes *vs* No	1.74 (0.68, 4.46)	0.25	—	—
ICI drugs	PD-L1 inhibitors *vs* PD-1 inhibitors	2.20 (1.11, 4.35)	0.02	2.37 (1.10, 5.11)	0.03
Stage	ED *vs* LD	3.20 (1.13, 9.03)	0.03	0.98 (0.29, 3.28)	0.97
ECOG PS	≥2 *vs* 0−1	6.30 (2.58, 15.36)	<0.001	6.96 (2.25, 21.55)	0.001
Brain metastasis	Yes *vs* No	1.83 (0.90, 3.71)	0.09	—	—
Liver metastasis	Yes *vs* No	4.58 (2.39, 8.78)	<0.001	2.66 (1.19, 5.93)	0.02
Bone metastasis	Yes *vs* No	5.61 (2.86, 10.97)	<0.001	4.61 (2.01, 10.59)	<0.001
Pretreatment LIPI	Intermediate/Poor *vs* Good	2.93 (1.54, 5.60)	0.001	2.34 (1.13, 4.86)	0.02

ICI, immune checkpoint inhibitor; PD-1, programmed cell death-1; PD-L1, programmed cell death-ligand 1; LD, limited disease; ED, extensive disease; ECOG PS, Eastern Cooperative Oncology Group Performance Status; LIPI, lung immune prognostic index; HR, hazard ratio; CI, confidence interval.

### Subgroup Analysis of Relationship Between LIPI and Survival Outcomes

**Figure 3:**
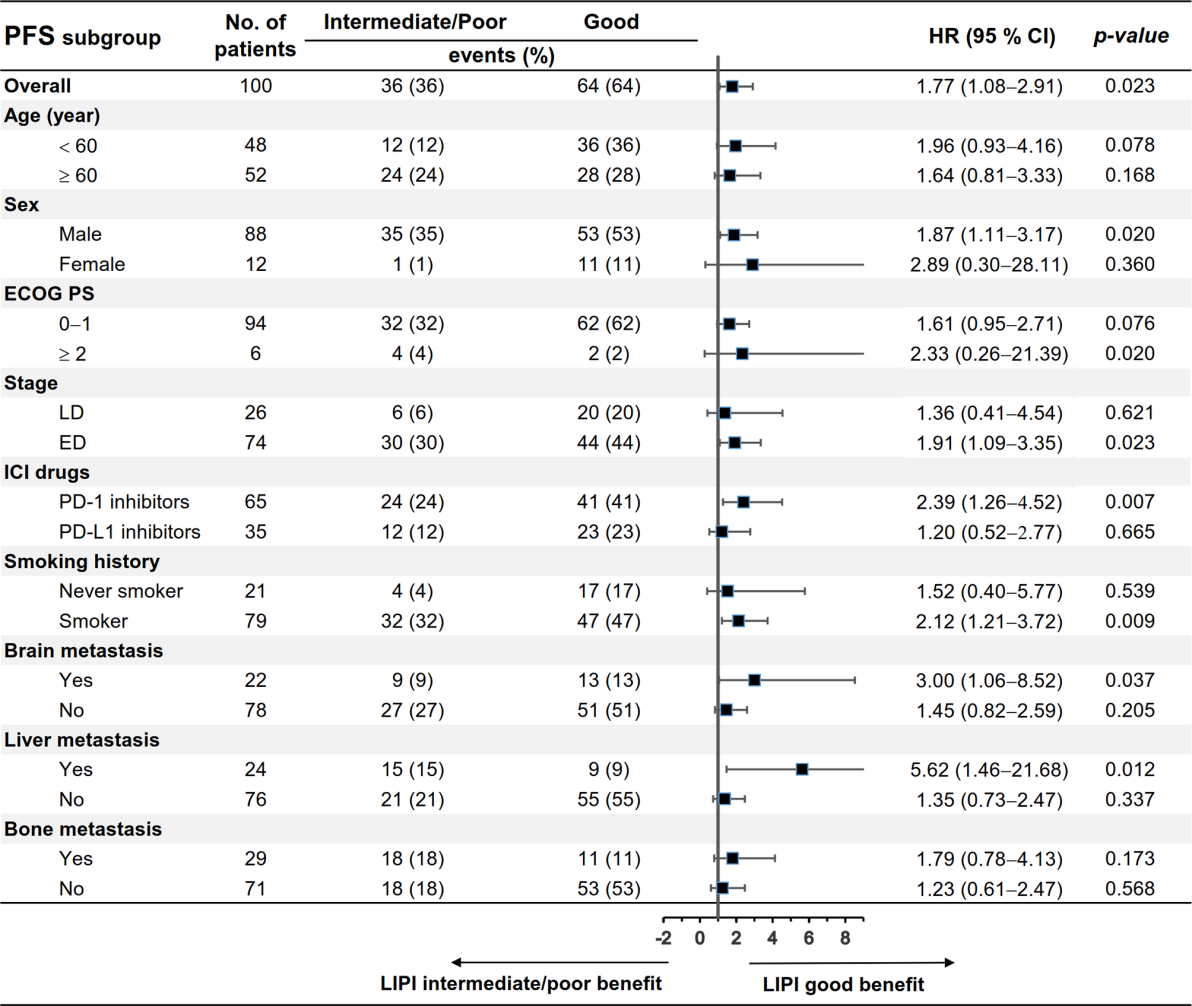
Subgroup analysis of the association between pretreatment LIPI and PFS.

**Figure 4:**
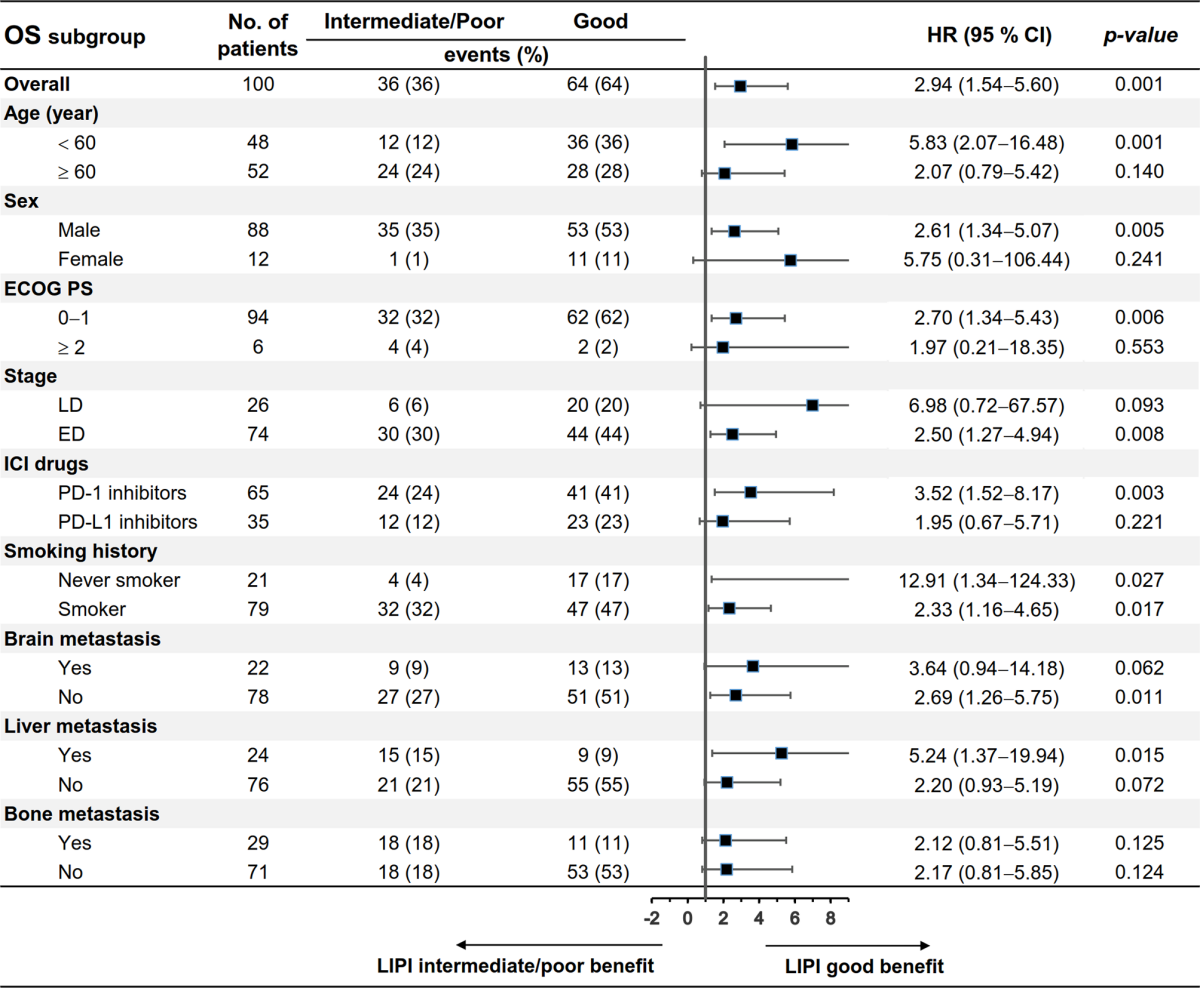
Subgroup analysis of the association between pretreatment LIPI and OS.

We evaluated the differences in patients’ characteristics between the LIPI good and LIPI intermediate/poor groups. The results indicated that age, liver metastasis, and bone metastasis were not balanced between the two groups (*p* < 0.05) (**Table 5**). Subgroup analysis stratified by these characteristics was further conducted. As shown in **Figures 3**, **4**, LIPI good was associated with better PFS and OS compared with LIPI intermediate/poor in males, smokers, those with ED, those receiving PD-1 inhibitors, and those with liver metastasis (*p* < 0.05).

**Table 5:** Differences of patients’ characteristics between the two groups.

Characteristics	Pretreatment LIPI		*p-value*
	Good	Intermediate/poor	
Age (year)			
<60	36	12	0.037
≥60	28	24	
Sex			
Male	53	35	0.051
Female	11	1	
Stage			
LD	20	6	0.154
ED	44	30	
Smoking history			
Never smoke	17	4	0.079
Smoke	47	32	
ICI drugs			
PD-1 inhibitors	41	24	0.83
PD-L1 inhibitors	23	12	
ECOG PS			
0−1	62	32	0.184
≥2	2	4	
Brain metastasis			
Yes	13	9	0.621
No	51	27	
Liver metastasis			
Yes	9	15	0.003
No	55	21	
Bone metastasis			
Yes	11	18	0.001
No	53	18	

ICI, immune checkpoint inhibitor; PD-1, programmed cell death-1; PD-L1, programmed cell death-ligand 1; LD, limited disease; ED, extensive disease; ECOG PS, Eastern Cooperative Oncology Group Performance Status; LIPI, lung immune prognostic index; HR, hazard ratio; CI, confidence interval.

## Discussion

Although ICIs have been established as an important option for treating patients with SCLC, these drugs are not beneficial for all patients. The method of selecting SCLC patients who could respond to immunotherapy remains unclear. Inflammatory markers have been found to be correlated with the survival of patients with lung cancer (27–34). The LIPI, calculated by dNLR and LDH, has been investigated as a prognostic factor for lung cancer. Mezquita et al. (23) reported that pretreatment LIPI was related to clinical outcomes of advanced NSCLC with ICI treatment, but not chemotherapy. However, Kazandjian et al. (35) demonstrated that LIPI was an important prognostic biomarker irrespective of treatment modality in NSCLC. Sonehara et al. (36) first revealed that LIPI could be used as a prognostic biomarker for SCLC patients, but the sample size was small, and the study involved patients without ICIs as first-line treatment. Other previous studies showed that pretreatment LIPI was a prognostic biomarker in ED-SCLC patients receiving chemotherapy or LD-SCLC patients (37, 38). In a recent retrospective study with data from a randomized clinical trial, inflammatory markers, including LIPI, were evaluated in ED-SCLC patients receiving atezolizumab and chemotherapy, and the results showed that LIPI was not an independent prognostic factor (39). However, their study had a small sample size and the patient population in the prospective clinical trial could not represent the entire SCLC population receiving first-line PD-1/PD-L1 inhibitor treatment.

To the best of our knowledge, this is the first study to demonstrate the relationship between pretreatment LIPI and the survival outcomes of SCLC patients receiving first-line ICI treatment. In previous studies, the included cohorts were divided into three groups (LIPI good, LIPI intermediate, and LIPI poor) (37–39). However, no obvious differences were reported between the LIPI intermediate and LIPI poor groups in terms of OS (37). In addition, few untreated patients had a poor LIPI score (11% patients in our study). Therefore, it might be more appropriate if the cohort was separated into two groups (LIPI good and LIPI intermediate/poor). In a previous study on the association of pretreatment LIPI with survival time in advanced hepatocellular carcinoma patients, the population was also divided into two groups (LIPI good and LIPI intermediate/poor) (40). Our findings showed that pretreatment LIPI was associated with PFS and OS in SCLC patients with first-line ICI treatment in univariate analysis. Multivariate analysis showed that pretreatment LIPI was an independent prognostic factor for OS, but not for PFS. However, the negative results of PFS should be interpreted with caution owing to the retrospective nature of this study. PFS was influenced by multiple factors, such as the frequency of evaluation of tumors. Conversely, the difference in OS between the LIPI good group and LIPI intermediate/poor group is more convincing. In addition, although multivariate analysis took many factors into consideration, other factors not included in the analysis, such as PD-L1, TMB and antibiotic therapy (41), may also affect the final results. We further conducted a subgroup analysis by patients’ characteristics, and the results indicated that the LIPI good group had better PFS and OS than the LIPI intermediate/poor group, especially in subgroups of males, smokers, those with ED, those receiving PD-1 inhibitor treatment, and those with liver metastasis, which revealed that the pretreatment LIPI might be prognostic only for specific subgroups of SCLC patients. However, these results need further investigation.

There were several limitations to this study. Firstly, it was a single-center retrospective study with a small sample size; therefore, some confounding factors and selective bias could not be avoided. Because the sample size of the LIPI poor group was too small, we divided the cohort into two groups (LIPI good and LIPI intermediate/poor) rather than three groups (LIPI good, LIPI intermediate, and LIPI poor) to conduct analyses. Secondly, considering the promising results of nivolumab plus chemotherapy as first-line treatment in SCLC patients in the EA5161 study and the accessibility and affordability of PD-L1 inhibitors in Chinese patients, 65% of the patients in this study were treated with PD-1 inhibitors, though only PD-L1 inhibitors have been approved as first-line treatment in SCLC patients by FDA. Thus, the interpretation of our results should be cautious due to drug selecting bias. Lastly, the cutoff values of dNLR and LDH were data-based and calculated using X-tile software, which may not have been optimal. Nevertheless, our study offered a simple and non-invasive method to help identify advanced SCLC patients who could benefit from first-line ICI plus chemotherapy treatment in clinical practice.

Our findings showed the prognostic value of pretreatment LIPI in advanced SCLC patients receiving first-line ICI treatment combined with chemotherapy, especially in males, those with ED, those receiving PD-1 inhibitor treatment, smokers, and those with liver metastasis. Pretreatment LIPI might serve as a useful tool to identify patients who may benefit from this treatment regimen.

## Data Availability Statement

The raw data supporting the conclusions of this article will be made available by the authors, without undue reservation.

## Ethics Statement

The study protocol was approved by the Ethics Committee of Chinese PLA general hospital. The patients/participants provided their written informed consent to participate in this study.

## Author Contributions

YH conceived the idea of this article. LL completed the work of acquisition of data. ZZ and LL shared the task of analysis, interpretation of data, and manuscript writing. All authors participated in discussing and revising the manuscript. All authors contributed to the article and approved the submitted version.

## Funding

This work was supported by the Military Health Special Research Project Under Grant 20BJZ37.

## Conflict of Interest

The authors declare that the research was conducted in the absence of any commercial or financial relationships that could be construed as a potential conflict of interest.

## Publisher’s Note

All claims expressed in this article are solely those of the authors and do not necessarily represent those of their affiliated organizations, or those of the publisher, the editors and the reviewers. Any product that may be evaluated in this article, or claim that may be made by its manufacturer, is not guaranteed or endorsed by the publisher.
